# Polymorphisms of the artemisinin resistant marker (K13) in *Plasmodium falciparum* parasite populations of Grande Comore Island 10 years after artemisinin combination therapy

**DOI:** 10.1186/s13071-015-1253-z

**Published:** 2015-12-15

**Authors:** Bo Huang, Changsheng Deng, Tao Yang, Linlu Xue, Qi Wang, Shiguang Huang, Xin-zhuan Su, Yajun Liu, Shaoqin Zheng, Yezhi Guan, Qin Xu, Jiuyao Zhou, Jie Yuan, Afane Bacar, Kamal Said Abdallah, Rachad Attoumane, Ahamada M. S. A. Mliva, Yanchun Zhong, Fangli Lu, Jianping Song

**Affiliations:** Science and Technology Park, Guangzhou University of Chinese Medicine, Guangzhou, 510006 Guangdong PR China; School of Medicine, Jinan University, Guangzhou, 510632 Guangdong PR China; Laboratory of Malaria and Vector Research, National Institute of Allergy and Infectious Diseases, National Institutes of Health, Bethesda, MD 20892 USA; State Key Laboratory of Cellular Stress Biology, Innovation Center for Cell Signaling Network, School of Life Sciences, Xiamen University, Xiamen, 361005 Fujian PR China; The first affiliated Hospital, Guangzhou University of Chinese Medicine, Guangzhou, 510006 Guangdong PR China; Research Institute of Tropical Medicine, Guangzhou University of Chinese Medicine, Guangzhou, 510006 Guangdong PR China; Traditional Chinese Medicine Collage, Guangzhou University of Chinese Medicine, Guangzhou, 510006 Guangdong PR China; National Malaria Control Programme, Moroni, Union of Comoros; Ministry of Health Comoros, Moroni, Union of Comoros; Department of Parasitology, Zhongshan School of Medicine, Sun Yat-sen University, Guangzhou, 510080 Guangdong PR China

**Keywords:** Comoros, *Plasmodium falciparum*, Artemisinin resistance, K13-propeller, Polymorphism

## Abstract

**Background:**

*Plasmodium falciparum* malaria is a significant public health problem in Comoros, and artemisinin combination therapy (ACT) remains the first choice for treating acute uncomplicated *P. falciparum*. The emergence and spread of artemisinin-resistant *P. falciparum* in Southeast Asia, associated with mutations in K13-propeller gene, poses a potential threat to ACT efficacy. Detection of mutations in the *P. falciparum* K13-propeller gene may provide the first-hand information on changes in parasite susceptibility to artemisinin. The objective of this study is to determinate the prevalence of mutant K13-propeller gene among the *P. falciparum* isolates collected from Grande Comore Island, Union of Comoros, where ACT has been in use since 2004.

**Methods:**

A total of 207 *P. falciparum* clinical isolates were collected from the island during March 2006 and October 2007 (*n* = 118) and March 2013 and December 2014 (*n* = 89). All isolates were analysed for single nucleotide polymorphisms (SNPs) and haplotypes in the K13-propeller gene using nested PCR and DNA sequencing.

**Results:**

Only three 2006–2007 samples carried SNPs in the K13-propeller gene, one having a synonymous (G538G) and the other having two non-synonymous (S477Y and D584E) substitutions leading to two mutated haplotypes (2.2 %, 2/95). Three synonymous mutations (R471R, Y500Y, and G538G) (5.9 %, 5/85) and 7 non-synonymous substitutions (21.2 %, 18/85) with nine mutated haplotypes (18.8 %, 16/85) were found in isolates from 2013 to 2014. However, none of the polymorphisms associated with artemisinin-resistance in Southeast Asia was detected from any of the parasites examined.

**Conclusion:**

This study showed increased K13-propeller gene diversity among *P. falciparum* populations on the Island over the course of 8 years (2006–2014). Nevertheless, none of the polymorphisms known to be associated with artemisinin resistance in Asia was detected in the parasite populations examined. Our data suggest that *P. falciparum* populations in Grande Comore are still effectively susceptible to artemisinin. Our results provide insights into *P. falciparum* populations regarding mutations in the gene associated with artemisinin resistance and will be useful for developing and updating anti-malarial guidance in Comoros.

## Background

Malaria remains one of the major public health problems throughout the world, with the occurrence of estimated 225 million clinical cases and ~600,000 deaths annually [1]. In the Union of Comoros, *Plasmodium falciparum* malaria is the most widespread species of human malaria parasites, and was responsible for 15 % ~ 20 % of the registered deaths historically [2]. Over the past six decades, chloroquine (CQ) had been used to cure and/or to prevent malaria in Comoros [3]. Nevertheless, the increasing emergence and spread of CQ-resistant *P. falciparum* led to the use of the combination sulfadoxine-pyrimethamine (SP) as first-line treatment for acute uncomplicated *P. falciparum* malaria in Comoros [3, 4]. Unfortunately, malaria control in Comoros had been imperiled by the emergence and spread of CQ- and SP-resistant *P. falciparum* in the early 1980s and of DDT-resistant *Anopheles* mosquitoes [5, 6]. In 2004, more than 100,000 malaria cases were reported in Comoros, with high level incidence (~35 %) [1]. Based on these observations, the government of Comoros introduced artemisinin combination therapy (ACT, artemether-lumefantrine) as first-line therapy for uncomplicated *P. falciparum* malaria, and officially withdrew the use of CQ and SP in 2004 [4, 5]. Unfortunately, the ACT regime did not lead to decrease of malaria incidence, with approximately 100,000 annual malaria cases recorded from 2006 to 2012. Thus, malaria still represents one of the major public health challenges in Comoros, being responsible for ~38 % out-patient consultations and ~60 % of all hospitalizations. A countrywide malaria control and elimination policy was launched by the government of Comoros in 2006, with the goal to basically control malaria by 2015 and to completely eliminate malaria by 2020. To block malaria transmission in this endemic area, mass drug administration with therapeutical dose of artemisinin-piperaquine and low-dose of primaquine (Artepharm Co. Ltd, PR China) was launched by late 2013 on Grande Comore Island. The effort has accelerated with impetus from the Global Fund in 2012, in which large-scale distribution of insecticide-treated mosquito nets has also been gradually implemented on Grande Comore, Moheli, and Anjouan Islands, Union of Comoros [4], hoping to reach up to 89.1 and 46.3 % of the households with at least 1 mosquito net and 1 insecticide-impregnated mosquito net, respectively. Consequently, the annual malaria cases dramatically changed from 114,537 in 2007 to 2142 in 2014 (a 98.0 % reduction). To achieve the goal of malaria elimination, there is an urgent need to monitor parasite susceptibility to artemisinin, and to develop and update anti-malarial guidance in Comoros.

ACT has been implemented world-wide as the first-line treatment for acute uncomplicated *P. falciparum* malaria since 2001, and has been responsible for the reduction in malaria-associated mortality and morbidity [7, 8]. Currently, artemisinin-resistant *P. falciparum* parasites have been reported mostly in Southeast Asia, including Cambodia, Thailand, Myanmar, Vietnam, and Laos [9–15]. Previous experience with the spread of CQ- and SP-resistant parasites from Asia to Africa suggests that the spread of artemisinin resistance to other parts of the world is likely, and that vigilant surveillance for artemisinin resistant parasites is important for controlling the spread of resistance [16–18]. Therapeutic efficacy studies under field conditions, considered the gold standard for determining anti-malarial drug efficacy, is currently complicated primarily due to limited numbers of malaria individuals available in low malaria transmission areas [19]. Therefore, molecular genetic markers of resistance are also useful for monitoring the emergence and spread of anti-malarial drug resistance [20, 21]. Mutations and variations in expression of several genes such as *pfmdr1* and *pfatpase6* have been suggested, but not proven, to be the molecular marker of artemisinin resistance [22–24]. Recent studies have shown that artemisinin resistance is associated with non-synonymous single-nucleotide polymorphisms (SNPs) in a *P. falciparum* gene with kelch propeller domain (K13-propeller, PF3D7_1343700) in Southeast Asia [12, 13, 25, 26]. Specifically, three mutations (C580Y, R539T and Y493H) in the K13-propeller gene were strongly associated with increased ring stage survival and delayed parasite clearance [12]. Meanwhile, artemisinin tolerance in vitro was associated with the M476I mutation of K13-propeller [12].

As resistance to earlier anti-malarial drugs (CQ and SP) spread from Asia to Africa and other parts of world through parasite migration, there is a serious concern that a similar scenario may occur with artemisinin resistance [16–18]. Recently, it was reported that highly artemisinin-resistant parasites could also emerge independently in Southeast Asia, indicating that K13-propeller gene polymorphisms have multiple origins throughout Africa as well as Southeast Asia [26]. Comoros is a malaria-endemic country with a history of ACT (artemether-lumefantrine) use since 2004, and a mass drug administration (MDA) of artemisinin-piperaquine plus low-dose of primaquine was launched for blocking malaria transmission on Grande Comore Island in 2013. Under such continuous and widespread artemisinin selective pressure, it can be expected that a similar phenomenon seen in Southeast Asia may occur in Comoros. Currently, no molecular epidemiologic information on *P. falciparum* parasite populations on Grande Comore Island in response to artemisinin is avaialable. Therefore, the aim of this study is to investigate the frequencies and patterns of mutations in K13-propeller gene linked to artemisinin resistance of Grande Comore *P. falciparum* clinical isolates. Our data will provide important information for molecular surveillance of artemisinin-resistant *P. falciparum* in this area.

## Methods

### Ethical statement

The study protocol was reviewed and approved by the Ethics Committees of Comoros Ministry of Health (protocol No. 12521/MSSPG/CAB) and Guangzhou University of Chinese Medicine (protocol No. 2012L0816). A written informed consent form was read and signed by all participants before any study procedure was performed. The individuals who could not read and write in the language, signed the form with their thumb print after it was completed by an independent witness on their behalf.

### Study sites

**Fig. 1:**
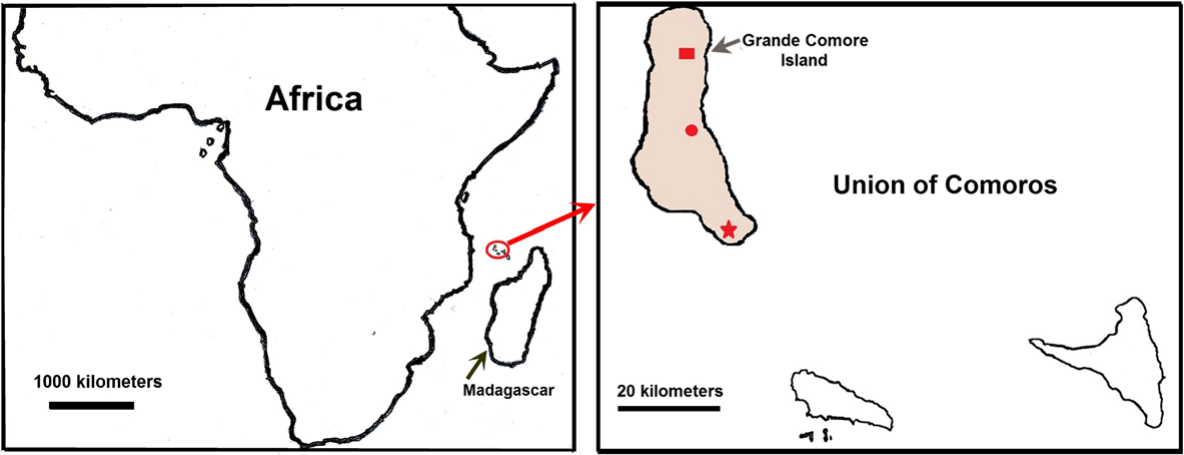
Map of Grande Comore Island, Union of Comoros. Shown are the locations of Mitsoudje Center Hospital (□), National Malaria Center (○), and Mitsamiouli Center Hospital (☆) where *P. falciparum* isolates were collected

The study was conducted on Grande Comore Island, Union of Comoros (latitude 11°00′-12°00′S; longitude 43°10′-43°35′E), in the Indian Ocean between Madagascar and the eastern coast of Africa (Fig. 1). This island has an area of 1147 km^2^ with a human population around 420,000 in 2012. The climate of this island is characterized by a warm, wet summer, and a cool, dry winter. The area has a distinct seasonal climate characterized by rainy season (November-April) and dry season (May-October), with temperature ranging from 11 to 35 °C and rainfall ranging from 1000 to 3000 mm annually. This island was a highly endemic area for *P. falciparum* and *P. malariae* malaria, with *P. falciparum* being the most common species (>95.5 %) [27]. Malaria transmission is perennial, with a peak during the rainy season throughout this island. Four confirmed *Anopheles* species (*Anopheles gambiae*, *An. funestus*, *An. merus*, and *An. coustani*) are present on this island, with *An. gambiae* being the predominant vector [27].

### Study samples

Blood samples were collected from patients with symptomatic *P. falciparum* malaria admitted to Mitsoudje Center Hospital, National Malaria Center, and Mitsamiouli Center Hospital for anti-malarial drug treatment (Fig. 1). After informed consent from all adults or legal guardians of children, thick and thin blood smears were prepared and were stained with 10 % Giemsa to diagnose human malaria species. Patients infected with *P. falciparum* only (no other human malaria species) as confirmed by peripheral smear examination were included in this study. One study was conducted between March 2006 and October 2007, and the other between March 2013 and December 2014. From each individual, a 1.0 ml of whole blood sample was collected in an EDTA tube and stored at −20 °C until DNA extraction. A total of 207 *P. falciparum* clinical isolates were collected on Grande Comore Island in one period March 2006 and October 2007 (*n* = 118), and in another period March 2013 and December 2014 (*n* = 89).

### Extraction of parasite DNA

Genomic DNA was extracted from 100 μL of each whole blood sample using DNA blood kit according to the manufacturer’s protocol (Takara, Japan). The extracted DNA was eluted in 60 μL of TE buffer (10 mM Tris–HCl, 0.1 M EDTA, pH 8.0) and stored at −20 °C until use. The quality of genomic DNA was detected using 1.0 % agarose gel electrophoresis and goldviewer buffer staining used as ethidium bromide substitute (Sangon Bio Inc., Shanghai, China).

### PCR amplification and sequencing of *P. falciparum* K13-propeller

To amplify the *P. falciparum* K13-propeller, a nested PCR amplification method was used following previously reported protocols [12]. Oligonucleotide primers and cycling conditions are listed in Table 1. The total 25 μl amplification reaction mixtures contained 8.5 ~ 10.0 μl of dH_2_O, 1.0 μl of each primer (10 pM), and 12.5 μl of *Taq* PCR Master Mix following the manufacturer’s instructions (Sangon Bio Inc., Shanghai, China). Primary amplification reactions were initiated with the addition of 2.0 μL of template genomic DNA prepared from the blood samples. For the nested PCR, 0.5 μL of primary PCR productions was used as template. The amplified PCR products were detected on 2 % agarose gel, and the sizes of the PCR products were measured visually based on a 100 bp DNA ladder (Sangon Bio Inc., Shanghai, China). The nested PCR products of K13-propeller were directly sequenced in both directions, using an ABI PRISM3730 DNA sequencer (Sangon Bio Inc., Shanghai, China). The nucleotide and amino acid sequences of K13-propeller were compared with wild-type amino acid sequence (GenBank accession number, XM_001350122) using Clustal W of the BioEdit 7.0 and MEGA 4.0 programs.

**Table 1:** Primer sequences and cycling conditions used to amplify K13-propeller gene of *Plasmodium falciparum isolates* ^a^

Genes^b^	Primers^c^	PCR cycling conditions	Product size	Reference
K13-propeller (P)	F: 5′-cggagtgaccaaatctggga-3′ R: 5′-gggaatctggtggtaacagc-3′	95 °C 5 min/[95 °C 30 s, 60 °C 90 s, 72 °C 90 s] × 40 cycles, 72 °C 10 min	2096	[12]
K13-propeller (S)	F: 5′-gccaagctgccattcatttg-3′ R: 5′-gccttgttgaaagaagcaga-3′	95 °C 5 min/[95 °C 30 s, 60 °C 90 s, 72 °C 90 s] × 40 cycles, 72 °C 10 min	848	[12]

^a^ *P. falciparum* isolates from Grande Comore Island, Union of Comoros

^b^ *P* Primary PCR reaction, *S* Secondary PCR reaction

^c^ *F* Forward primer, *R* Reverse primer

### Statistical analysis

Statistical significance was determined with SPSS software (version 13.0). Mann–Whitney *U* test was used to compare in the prevalence of the mutations and alleles of parasite samples between two collected periods. Statistical significance was set at *P* < 0.05.

## Results

All blood samples (*n* = 207) were collected from *P. falciparum* mono-species infections identified after microscopy examination of blood smears. Because of low parasitemia and/or poor DNA quality, we were not able to successfully amplify the *P. falciparum* K13-propeller gene from all the samples; only 87.0 % of the *P. falciparum* isolates from Grande Comore Island (*n* = 180) could be amplified, including samples collected during 2006–2007 (*n* = 95) and 2013–2014 (*n* = 85). Sequencing of the gene revealed only one synonymous mutation at codons G538G (GGT → GGA) among the isolates from 2006 to 2007 group (1.1 % of the isolates examined, 1/95) (Table 2). Three synonymous substitutions, R471R (CGT → CGC), Y500Y (TAT → TAC), and G538G (GGT → GGA) were found in isolates from 2013 to 2014 (5.9 % of the isolates examined, 5/85). Interestingly, G538G substitution was detected in isolates from both groups. Meanwhile, non-synonymous mutations at codons S477Y and D584E, with the frequencies of 2.2 % (2/95), were detected in isolates from 2006 to 2007. In contrast, more than 20 % (18/85) of the isolates from 2013 to 2014 carried seven non-synonymous mutations (D464H, D464Y, S477Y, L488S, A504T, I526M, A578S, and D584E) in the K13-propeller domain, including two mutations (S477Y and A578S) that have been reported previously in Africa (Kenya and Uganda). Of these mutations, the most prevalent change was D464H/Y (5.9 %, 5/85), followed by A578S (4.7 %, 4/85) and D584E (3.5 %, 3/85). The number of non-synonymous mutations increased significantly from 2.2 % (in 2006–2007) to 21.2 % (in 2013–2014) (*P* < 0.05) in Grande Comore isolates. However, none of the C580Y, R539T, or Y493H mutations previously associated with delayed parasite clearance in Southeast Asia isolates, or the M476I substitution selected in vitro in a Tanzanian strain, was observed in these parasite populations (Table 2).

**Table 2:** Prevalence of K13-propeller mutations in *Plasmodium falciparum* isolates^a^

Areas	Mutations	Amino acid and genetic changes^b^	Number of isolates (%)^c^	
			2006–2007 (*n* = 95)	2013–2014 (*n* = 85)
Mitsoudje Center Hospital	Synonymous	R471**R** (CGT → CG**C**)	0 (0)	1 (1.2)
		G538**G** (GGT → GG**A**)	1 (1.1)	2 (2.4)
	Non-synonymous	D464**H** (GAT → **C**AT)	0 (0)	1 (1.2)
		D464**Y** (GAT → **T**AT)	0 (0)	1 (1.2)
		L488**S** (TTG → T**C**G)	0 (0)	1 (1.2)
		I526**M** (ATA → AT**G**)	0 (0)	1 (1.2)
		A578**S** (GCT → **T**CT)	0 (0)	2 (2.4)
		D584**E** (GAT → GA**A**)	1 (1.1)	1 (1.2)
National Malaria Center	Synonymous	Y500**Y** (TAT → TA**C**)	0 (0)	1 (1.2)
	Non-synonymous	D464**H** (GAT → **C**AT)	0 (0)	1 (1.2)
		S477**Y** (TCT → T**A**T)	0 (0)	1 (1.2)
		I526**M** (ATA → AT**G**)	0 (0)	1 (1.2)
		A578**S** (GCT → **T**CT)	0 (0)	2 (2.4)
Mitsamiouli Center Hospital	Synonymous	Y500**Y** (TAT → TA**C**)	0 (0)	1 (1.2)
	Non-synonymous	D464**Y** (GAT → **T**AT)	0 (0)	2 (2.4)
		S477**Y** (TCT → T**A**T)	1 (1.1)	1 (1.2)
		A504**T** (GCT → **A**CT)	0 (0)	1 (1.2)
		D584**E** (GAT → GA**A**)	0 (0)	2 (2.4)

^a^Isolates collected from 2006 to 2007 and 2013 to 2014 along Grande Comore Island of Comoros

^b^The mutated amino acids and nucleotides are indicated in bold type

^c^Statistically significant differences for comparison with isolates circulating in 2006–2007 from Grande Comore Island (*P < 0.05) using Mann–Whitney *U* test

Haplotype analysis of *P. falciparum* K13-propeller gene in isolates from 2006 to 2007 revealed only three distinct allelic forms (Table 3), including the wild-type allele, and two single-mutant alleles (477Y and 584E). Of the three allelic variants, the most prevalent allelic variant was wild-type allele (97.9 %, 93/97). Ten allelic variants [wild-type allele, seven single-mutant alleles (464H, 464Y, 477Y, 488S, 526M, 578S, and 584E), and two double-mutant alleles (477Y/504T and 526M/578S)] were detected in 85 isolates from 2013 to 2014, with wild-type allele being predominant (81.2 %, 69/85). The remaining allelic variants were evenly distributed at low frequency (1.2 to 3.5 %) among isolates from 2013 to 2014. It should be noted that all three haplotypes [(wild-type allele, and two single-mutant alleles (477Y and 584E)] detected in 2006–2007 isolates were also found in 2013–2014. Over the course of 8 years (2006–2014), the wild-type allele prevalence significantly decreased from 97.9 to 81.2 % (*P* < 0.05), and the mutant alleles prevalence significantly increased from 2.1 to 18.8 % (*P* < 0.05) in Grande Comore isolates. However, no significant differences in each measured mutated allelic forms were found between 2006–2007 and 2013–2014 groups (*P* > 0.05).

**Table 3:** Prevalence of single nucleotide polymorphisms and multi-mutated haplotypes in *Plasmodium falciparum* K13-propeller gene^a^

Areas	Genotypes	Number of isolates (%)^b^	
		2006–2007 (*n* = 95)	2013–2014 (*n* = 85)
Mitsoudje Center Hospital	Wild-type haplotype D_464_S_477_L_488_A_504_I_526_A_578_D_584_	30 (31.6)	20 (23.5)*
	Single-mutant haplotype **H**_464_S_477_L_488_A_504_I_526_A_578_D_584_	0 (0)	1 (1.2)
	Single-mutant haplotype **Y**_464_S_477_L_488_A_504_I_526_A_578_D_584_	0 (0)	1 (1.2)
	Single-mutant haplotype D_464_S_477_ **S**_488_A_504_I_526_A_578_D_584_	0 (0)	1 (1.2)
	Single-mutant haplotype D_464_S_477_L_488_A_504_I_526_ **S**_578_D_584_	0 (0)	1 (1.2)
	Single-mutant haplotype D_464_S_477_L_488_A_504_I_526_A_578_ **E**_584_	1 (1.1)	1 (1.2)
	Double-mutant haplotype D_464_S_477_L_488_A_504_ **M**_526_ **S**_578_D_584_	0 (0)	1 (1.2)
National Malaria Center	Wild-type haplotype D_464_S_477_L_488_A_504_I_526_A_578_D_584_	45 (47.4)	33 (38.9)*
	Single-mutant haplotype **H**_464_S_477_L_488_A_504_I_526_A_578_D_584_	0 (0)	1 (1.2)
	Single-mutant haplotype D_464_ **Y**_477_L_488_A_504_I_526_A_578_D_584_	0 (0)	1 (1.2)
	Single-mutant haplotype D_464_S_477_L_488_A_504_ **M**_526_A_578_D_584_	0 (0)	1 (1.2)
	Single-mutant haplotype D_464_S_477_L_488_A_504_I_526_ **S**_578_D_584_	0 (0)	2 (2.4)
Mitsamiouli Center Hospital	Wild-type haplotype D_464_S_477_L_488_A_504_I_526_A_578_D_584_	18 (18.9)	16 (18.8)
	Single-mutant haplotype **Y**_464_S_477_L_488_A_504_I_526_A_578_D_584_	0 (0)	2 (2.4)
	Single-mutant haplotype D_464_ **Y**_477_L_488_A_504_I_526_A_578_D_584_	1 (1.1)	0 (0)
	Single-mutant haplotype D_464_S_477_L_488_A_504_I_526_A_578_ **E**_584_	0 (0)	2 (2.4)
	Double-mutant haplotype D_464_ **Y**_477_L_488_ **T**_504_I_526_A_578_D_584_	0 (0)	1 (1.2)

Note: The double mutants were confirmed in repeated amplification and sequencing experiments

^a^In isolates collected from 2006 to 2007 and 2013 to 2014 along Grande Comore Island, Union of Comoros. The mutated amino acids are indicated by bold type

^b^Statistically significant differences for comparison with isolates circulating in 2006–2007 from Grande Comore Island (*P < 0.05) using Mann–Whitney *U* test

## Discussions

In Comoros, ACT has been introduced as the first-line treatment for uncomplicated *P. falciparum* malaria since 2004 [4, 5]. Although ACT remains highly efficacious for the treatment of falciparum malaria, and delayed parasite clearance of ACT has not been noted in Comoros [28], it is important to monitor the potential presence of artemisinin-resistant *P. falciparum* populations because the emergence and spread of artemisinin-resistant strains in Southeast Asia-an epicenter of resistance to several other anti-malarial drugs (CQ and SP) [9–15, 29]. Clinical artemisinin resistance is defined as a reduced parasite clearance rate, expressed as an increased parasite clearance half-life or a persistence of microscopically detectable parasites on the third day of ACT. The half-life parameter correlates strongly with results from the in vitro ring-stage survival assay (RSA_0–3 h_) and results from the ex vivo RSA. Unfortunately, due to poor field conditions (frequent power outage and the lack of liquid N_2_ storage and in vitro parasite culture), we were not able to culture *P. falciparum* parasites collected from Grande Comore Island for RSA_0–3_ assay. At present, we are not sure whether the point mutations in K13-propeller gene can serve as molecular markers for detecting potential artemisinin resistance and for monitoring malaria-control measures in Comoros. This study investigated the prevalence of mutant SNPs in K13-propeller gene of Grande Comore *P. falciparum* isolates collected from two different periods (2006–2007 and 2013–2014). Our data indicated that the frequencies of non-synonymous mutations in K13-propeller significantly increased in isolates from 2013 to 2014 when compared with those from 2006 to 2007. However, none of the mutations (C580Y, R539T, Y493H, and M476I) previously associated with artemisinin resistance in Southeast Asia parasites [12, 13, 25, 26] was observed in the parasite populations collected from both examined periods, suggesting that *P. falciparum* populations in Grande Comore are still effectively susceptible to artemisinin.

Our data were in line with other reports describing K13-propeller polymorphisms in *P. falciparum* collected from Africa, in which 24 non-synonymous mutations were detected at low frequencies, and most of them were different from the mutations observed in Southeast Asia when more than 2600 samples were analyzed from 15 countries [30–32]; Meanwhile, with the exception of 1 case in Nigeria and 2 cases in Congo, there were no reported cases of delayed parasite clearance or of prolonged RSA_0–3_ hours in sub-Saharan Africa to date [13, 33]. These observations suggest that not all K13-propeller mutations are associated with artemisinin resistance, and that secondary loci are involved in resistance Asian parasites, but not in African parasites. Additionally, high levels of antimalarial immunity can mask a resistance phenotype [34]. Unlike in Southeast Asia, individuals in malaria-endemic regions of sub-Saharan Africa are less likely to be infected with clonal parasites because of the high transmission intensity and are thus likely to lose potential resistance-conferring alleles to outcrossing. It is known that the subpopulations of parasites can be categorized as artemisinin-susceptible or -resistant, based on their genetic profile [35]. Therefore, the different polymorphisms in K13-propeller gene observed between Southeast Asia and Africa may be due to different genetic backgrounds. Whether the K13-propeller SNPs we observe here can be used as molecular markers for artemisinin resistance globally requires further investigations considering various parameters such as malaria parasite ancestry, geographical, clinical, epidemiological, and genetic diversities [31].

This study identified three synonymous mutations (R471R, Y500Y, and G538G) in K13-propeller in 3.3 % (6/180) of examined isolates. Of these mutations, mutation at codon G538G was detected in both examined periods (2006–2007 and 2013–2014) with the similarly low frequency (less than 3 %), indicating that the emergence of parasites with synonymous mutation at codon G538G should not attribute to artemisinin selective pressure on Grande Comore Island. In the present study, two synonymous mutations (Y500Y and G538G) were reported from parasite population in Kenya [36]. Similarly, R471R synonymous mutation found in this study was observed in Congo [30], Gabon [31], and Angola [37]. Although these synonymous mutations should not affect K13-propeller protein structure and function and likely plays no role in artemisinin resistance, the paucity of shared synonymous mutation between Comoros’ and African parasites suggesting that there exists a large reservoir of K13-propeller polymorphisms globally.

It has been suggested that Y493H, I543T, R539T, and C580Y mutations in K13-propeller gene are associated with prolonged parasite survival ex vivo; Y493H, R539T, and C580Y mutations are linked to in vivo delayed parasite clearance; and M476I mutation is related to artemisinin tolerance in vitro [12, 13, 25, 26]*.* In the present study, only two mutations at codons S477Y (1.1 %) and D584E (1.1 %) and two mutated-allelic types were detected in isolates from 2006 to 2007, suggesting that *P. falciparum* isolates collected in 2006–2007 showed very limited variability within the K13-propeller gene. In contrast, mutations at positions 464 (5.9 %), 477 (2.4 %), 488 (1.2 %), 504 (1.2 %), 526 (2.4 %), 578 (4.7 %), and 584 (3.5 %) leading to nine mutated allelic types (18.8 %) in K13-propeller gene were detected in isolates from 2013 to 2014. Our data was similar to those in other reports, where three of the 18 imported malaria isolates (16.7 %) from the Grande Comore showed polymorphism (N490H, N554K and E596G), and double-mutant haplotype occurred at very low frequencies [38]. The data in the current study showed that a trend of increase in the number of polymorphismic sites in *P. falciparum* K13-propeller gene is presented in Grande Comore isolates through the years of sample collection, may be due to the selective artemisinin pressure continually from the long history of ACT use since 2004. However, mutations strongly associated with artemisinin resistance (C580Y, R539T, Y493H, and M476I) in Southeast Asia parasites were not observed in isolates from both examined periods in the present study. Our observation is consistent with those in previous reports from Kenya [36], Angola [37], Mozambique [37], Senegal [39], Ugandan [40] as well as other areas of Sub-Saharan Africa [30], Caribbean’s Haiti [41], and South Asia’ Bangladesh [42], where the K13-propeller gene mutations associated with artemisinin resistance were absent. Additionally, trials of ACT for treating uncomplicated *P. falciparum* malaria in Uganda [43], Kenya [44], and Mozambique [45] have found 28-day PCR-corrected cure rates of 89 % or greater. Thus, our data is strongly suggesting that many Grande Comore *P. falciparum* isolates collected in 2013–2014 are likely still sensitive to artemisinin even under the drug selective pressure from introduction of ACT as the first-line treatment since 2004. Additionally, our results also suggest that selective artemisinin drug pressure may exist in Grande Comore Island that promotes these mutations, but not substantially enough to lead clinical drug resistance. Whether or not the non-synonymous substitutions in K13-propeller gene observed in our study are associated with artemisinin drug response, can not be inferred from our results. However, these mutations in K13-propeller gene observed in our study may provide some background mutations setting the stage for emergence of artemisinin resistance. Studies of careful comparison of parasites with these allelic types and their response to artemisinin are necessary to determine whether these mutations contribute artemisinin resistance.

Recently, it was reported that highly artemisinin-resistant parasites could have emerged independently in Southeast Asia [26]. In the present study, seven non-synonymous mutations were detected among the 180 Grande Comore isolates. Of these mutations, five occurred at positions 464, 488, 504, 526, and 584. These mutations present new mutations that have not been previously reported in other endemic areas, suggesting that independent emergence of the same mutations may occur on Grande Comore Island. Notably, the S477Y mutation detected in the present study was comparable to those reported in other areas of the world, such as Kenya [36]. Similarly, the A578S mutation detected in Grande Comore Island isolates was concordance with previous reports in Kenya [36], Uganda [30, 40], South Asia’ Bangladesh [42] and Cambodia [12]. Therefore, our data suggested that these mutations in K13-propeller gene of *P. falciparum* populations have a global distribution. But it remains an open question as to whether the presence of A578S mutation in these samples was due to independent emergence or had spread from other areas. Protein modeling data suggest that A578S can alter the function of the K13 propeller protein because it lies adjacent to the C580Y mutation that can cause delayed parasite clearance in Cambodia [42]. Nevertheless, the artemisinin collaboration group also found the A578S mutation in one specimen within their study areas but this mutation was not associated with increased parasite clearance half-life [12]. In addition, other mutation (V566I) is also located close to the C580Y mutation [12, 31]; however, whether V566I mutation has the same ability to alter the function of K13 protein required further investigation. Due to the presence of many different mutations among parasites from various endemic regions, further studies are required to validate the effect of the mutations.

## Conclusion

Results from the current study showed that the number of mutations in the *P. falciparum* K13-propeller gene has significantly increased in Grande Comore isolates from 2006 to 2014. However, none of the mutations (C580Y, R539T, Y493H, or M476I) previously associated to artemisinin resistance in Southeast Asia parasites was observed in parasite populations collected from the Grande Comore Island (2006–2007 and 2013–2014). Although we were not able to collect data related to the in vivo or in vitro efficacy of ACT on parasites in this study, the results suggest that *P. falciparum* populations on Grande Comore Island are still susceptible to artemisinin, even under widespread drug selective pressure from the introduction of ACT as the first-line treatment for over 10 years. Careful monitoring of parasite populations on changes in the K13-propeller gene and genes known to play a role in resistant to other antimalarial drugs are necessary.
